# Viewing Pain and Happy Faces Elicited Similar Changes in Postural Body Sway

**DOI:** 10.1371/journal.pone.0104381

**Published:** 2014-08-05

**Authors:** Juan Gea, Miguel A. Muñoz, Isis Costa, Luís F. Ciria, José G. V. Miranda, Pedro Montoya

**Affiliations:** 1 Research Institute on Health Sciences (IUNICS), University of the Balearic Islands, Palma de Mallorca, Spain; 2 Department of Personality, University of Granada, Granada, Spain; 3 Department of Physics of the Earth and the Environment, Federal University of Bahia, Salvador, Brazil; University of Tuebingen Medical School, Germany

## Abstract

Affective facial expressions are potent social cues that can induce relevant physiological changes, as well as behavioral dispositions in the observer. Previous studies have revealed that angry faces induced significant reductions in body sway as compared with neutral and happy faces, reflecting an avoidance behavioral tendency as freezing. The expression of pain is usually considered an unpleasant stimulus, but also a relevant cue for delivering effective care and social support. Nevertheless, there are few data about behavioral dispositions elicited by the observation of pain expressions in others. The aim of the present research was to evaluate approach–avoidance tendencies by using video recordings of postural body sway when participants were standing and observing facial expressions of pain, happy and neutral. We hypothesized that although pain faces would be rated as more unpleasant than the other faces, they would provoke significant changes in postural body sway as compared to neutral facial expressions. Forty healthy female volunteers (mean age 25) participated in the study. Amplitude of forward movements and backward movements in the anterior-posterior and medial-lateral axes were obtained. Statistical analyses revealed that pain faces were the most unpleasant stimuli, and that both happy and pain faces were more arousing than neutral ones. Happy and pain faces also elicited greater amplitude of body sway in the anterior-posterior axes as compared with neutral faces. In addition, significant positive correlations were found between body sway elicited by pain faces and pleasantness and empathic ratings, suggesting that changes in postural body sway elicited by pain faces might be associated with approach and cooperative behavioral responses.

## Introduction

According to Lang's bio-informational model [1], emotions are dispositions to action and reflect the activation of approach or avoidance behavior [2]. Basically, approach behaviors are elicited by pleasant and activating stimuli such as food, nurturance or caregiving, whereas avoidance behaviors are elicited by unpleasant and activating stimuli such as dangerous, threatening situations or painful experiences [3], [4]. In natural situations, approach and avoidance behaviors are closely correlated with emotional responses and, therefore, affective facial expressions may influence our social interactions [1], [5], [6].

Functional brain imaging research has corroborated that facial expressions are processed in specific brain regions correlated with emotional and empathic responses [7], [8]. Moreover, several studies have found that facial expressions are “contagious”, showing that viewing angry faces increases activity from the *corrugator supercilii* muscle, whereas viewing happy faces increases activity from the *zygomatic major* muscle [9], [10]. Accordingly, it has been suggested that because facial expressions are potent emotional stimuli that communicate information to the observer, they could predispose to approach and avoidance behaviors [11], [12]. In this sense, it has been shown that participants reacted faster to angry than to fear faces when they were asked to move forward or backward from affective stimuli by either flexing or extending their arms [6]. Similarly, it seems that participants displayed more approaching movements in response to happy and sadness faces and more avoiding movements in response to disgust and angry faces [13]–[15]. These results seem to indicate that facial expressions as happiness, fear or sadness are correlated with approach responses (such as help request or an invitation to cooperate), while disgust and anger faces are more correlated with avoidance responses.

Regarding approach-avoidance motor responses to affective stimulation, most studies examined arm movements or steps forward/backward as overt reactions [15]–[17]. Recently, several studies have focused on other behavioral measures such as postural sway [18]–[24]. The results of these studies indicated that participants displayed increased forward sway movements of the body when they stand on a force platform viewing pleasant pictures, but increased backward sway movements when they are viewing unpleasant pictures [25]–[27]. Basically, these findings support the notion of a relationship between motivated affective reactions and approach-avoidance behavior [13].

To our knowledge, there is however only one recent study examining body sways in response to affective facial expressions (angry, neutral and happy) when standing on a stabilometric force platform [28]. Although analyses of postural changes did not reveal approach or avoidance responses to any facial expressions, the authors found that angry faces induced significant reductions in body sway as compared with neutral faces and happy faces. The aim of the present study was to evaluate approach-avoidance behavior when participants were watching facial expressions of pain. Signaling pain in others is highly salient [29], [32], [33] and elicits empathic responses including changes in observers facial expression and vicarious pain [36]. Therefore, we hypothesized that pain faces would elicit significant differences in body sway as compared to neutral and happy facial expressions.

## Materials and Methods

### Ethics Statement

The study procedure was according with the Declaration of Helsinki principles and was approved by the Clinical Research Ethics Committee of the Balearic Islands (Spain) (protocol reference IB833/07 PI).

### Participants

The sample consisted of 40 female university students between 18 and 34 years (mean = 25, SD = 3.2). Prospective participants who reported hearing difficulties, visual or motor impairments (e.g., uncorrected vision or postural dysfunction), psychiatric problems, or on-going medical, psychological treatment and taking medication were excluded from the study. Participants received course credits for their participation.

### Self-report measures

After the written consent form was signed, participants completed the Interpersonal Reactivity Index (IRI) [30]. This questionnaire was originally designed to assess empathy from a multidimensional perspective and consists of 28 items distributed in four subscales measuring the cognitive and affective dimensions of empathy: Perspective Taking (PT), assessing the tendency to adopt the psychological viewpoint of others; Fantasy (FS), measuring the tendency to identify oneself emotionally with characters in fictional situations; Empathic Concern (EC), assessing the tendency to experience feelings of warmth and concern for others; and Personal Distress (PD), measuring self-oriented feelings as a result of witnessing another's emotional distress.

Participants also completed the Self-Assessment Manikin (SAM) [31] to rate pleasantness and arousal elicited by affective facial expressions. This instrument consists of two sets of humanoid figures representing the dimensions of pleasantness and arousal. Each rating scale includes nine levels of intensity, ranging from a smiling to a frowning figure for pleasantness and from an apparently agitated to a sleepy-looking figure for arousal. Participants were instructed to assess how they felt while viewing each facial expression by using these scales.

### Experimental task

**Figure 1:**
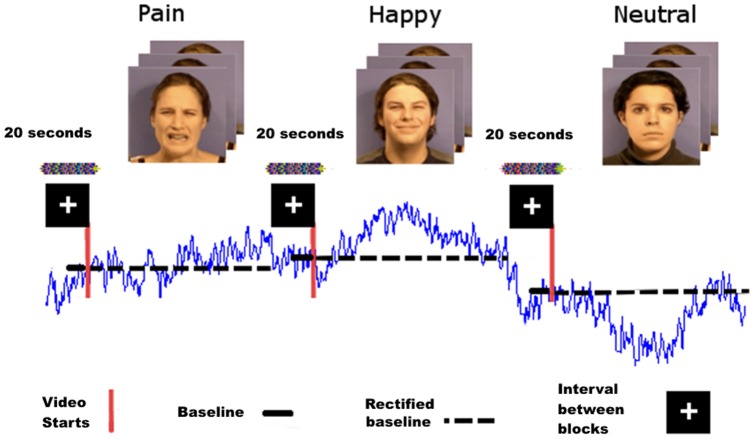
Description of experimental task and body sway signals elicited when viewing different facial expressions. Data were rectified by subtracting the mean of body sway during the first second before starting the presentation of each block of facial expressions. Data above the baseline represent the amplitude of forward movements and data below the baseline correspond to the amplitude of backwards movements.

The experimental task was similar to that used by Roelofs and colleagues [28] to examine behavioral responses to affective facial expressions (happy, neutral, and angry). Unlike the previous study, we decided to use dynamic rather than static facial expressions and to include expressions of pain instead of angry. For this purpose, stimuli were taken from a set of video clips showing different facial expressions developed and validated by Simon and colleagues [29], and already used in previous studies of our lab [32], [33]. In the present study, eight faces of four males and four females displaying neutral, happy and pain facial expressions were used as affective stimuli. Original video clips displayed individual expressions starting with a neutral face and ending at the peak of each expression for 1 second. In order to obtain a similar presentation time of the facial expressions as in Roelof et al's study [28], original video clips were slowed down to 2.5 seconds length and presented consecutively in blocks of 30 video clips with the same facial expressions (75 seconds) (Figure 1). Blocks were timely separated by a 20-seconds black screen with a white fixation cross. The presentation order of blocks was pseudorandomized across subjects by using a Latin square design. The task always started with a 60-seconds black screen with a white fixation cross to stabilize subject's body sway.

The video clips were displayed by using the ePrime V2.0 software in a 38″ screen situated at a distance of 200 cm and with view angles of 23° (horizontal) and 17° (vertical). In order to record participant movements, an electronic led diode was located on top of the head and small changes on postural body sway were measured by a webcam located few centimeters above the head. Participants were asked to remain standing with their arms hanging alongside their body and positioned in their normal, comfortable stance without sharp movements and relaxed when observing the stimuli. The task was performed in a small, dimly lit room.

At the end of the experiment, each video was presented again followed by a 15 second interval and subjects were instructed to rate pleasantness and arousal elicited by facial expressions.

### Video recording and data acquisition

Body sway was recorded with a standard webcam (©Logitech) at 30 frames per second located above the subject and connected to a laptop. Video recordings were analyzed by extracting data about trajectories in anterior-posterior and medial-lateral axes generated from postural adjustments with an open-source software (CvMob 3.1, http://www.cvmob.ufba.br/) [34]. The led diode located on subject's head was used as reference marker.

Data were analyzed offline using a Matlab program that rectified the signal from anterior-posterior and medial-lateral axes subtracting the mean of sway for one second baseline prior to the presentation of each block (Figure 1). Following parameters were obtained from video recordings to characterize body sways:

- magnitude of body sway in the anterior–posterior axis, defined as the standard deviation of displacement in this axis;
- magnitude of body sway in the medial–lateral axis, defined as the standard deviation of displacement in this axis;
- amplitude of forward movements in the anterior-posterior axis, defined as the amount of displacement accumulated above the reference marker (forward area);
- amplitude of backward movements in the anterior-posterior axis, defined as the amount of displacement accumulated below the reference marker (backward area).

### Statistical analysis

Subjective and body sway data were initially checked for normal distribution by using the Shapiro-Wilk test. Because our datasets significantly deviated from a normal distribution, the effects of facial expression (happy, neutral and pain) on dependent variables were assessed by using the Friedman test. In addition, the Wilcoxon signed-rank test was used to compare all pairs of levels of the independent variable (facial expression). In order to further explore the relationship between subjective data (pleasantness, arousal, and empathy) and body sway elicited by facial expressions of pain, happy and neutral, Kendalls' rank correlations were computed among these variables separated for each level of the independent variable. All analyses were performed with SPSS 19.0 statistical package. A significance level of p = .05 was used for all statistical analyses.

## Results

### Subjective ratings

The Friedman test yielded a significant effect of facial expressions on pleasantness [χ^2^(2) = 65.59, p<.001] and arousal ratings [χ^2^(2) = 12.02, p<.01]. Table 1 shows mean and standard deviation of pleasantness and arousal ratings for each facial expression. Post-hoc pairwise mean comparisons revealed that pain faces were more unpleasant than both neutral [Z = 4.81, p<.001] and happy faces [Z = 5.41, p<.001], and that happy faces were also more pleasant than neutral ones [Z = 4.87, p<.001]. In addition, pain faces were more arousing than neutral faces [Z = 3.19, p<.01], whereas there were no differences on arousal ratings between pain and happy faces [Z = 1.04, NS], or between happy and neutral faces [Z = 1.81, NS].

**Table 1:** Mean (and standard deviation) of pleasantness and arousal ratings elicited by pain, happy and neutral faces.

	Pleasantness	Arousal
Pain	2.75 (1.1)	5.47 (2.7)
Neutral	4.60 (1.3)	4.05 (2.4)
Happy	7.57 (1.4)	5.02 (2.5)

Pleasantness ratings ranged from 1 (very unpleasant) to 9 (very pleasant), and arousal ratings range from 1 (very calm) to 9 (very excited).

### Postural sway of the body

**Figure 2:**
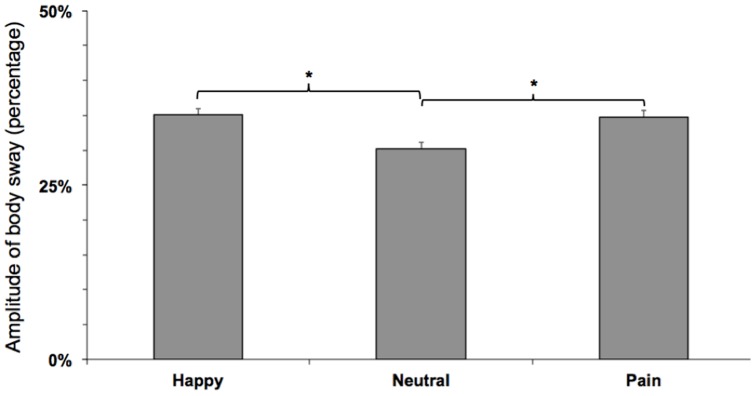
Amplitudes of postural body sway in the anterior-posterior axis elicited by viewing facial expressions and expressed in percentage of change with respect to baseline. Asterisks indicate significant differences at 5% level.

The Friedman test yielded significant effects of facial expressions on body sway in the anterior–posterior axis [χ^2^(2) = 9.05, p<.01]. Post-hoc pairwise mean comparisons revealed that happy and pain faces elicited higher body sway than neutral faces [happy vs. neutral faces: Z = 3.02, p<.01; pain vs. neutral faces: Z = 2.61, p<.01], whereas no difference was yielded between pain and happy faces [Z = .24, NS] (Figure 2).

**Figure 3:**
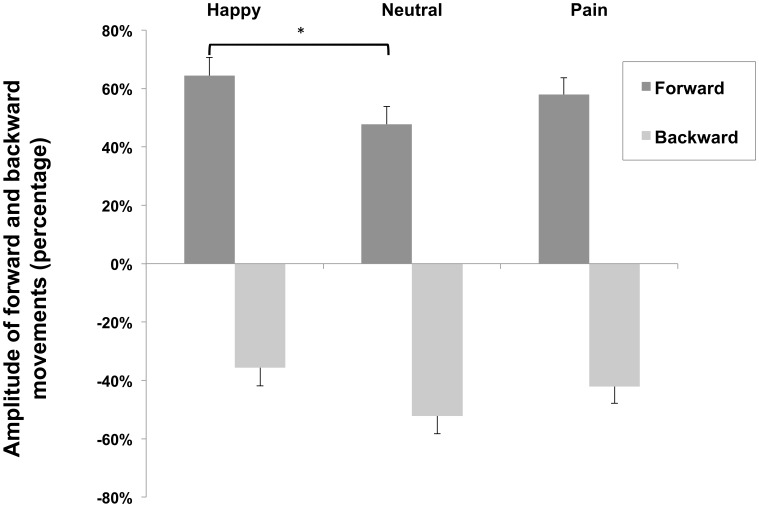
Amplitudes of forward and backward movements elicited by viewing facial expressions and expressed in percentage of change with respect to baseline. Positive values refer to forward and negative values to backward movements. Asterisks indicate significant differences at 5% level.

The Friedman test also revealed significant effects of facial expression on the amplitude of forward body movements [χ^2^(2) = 6.05, p<.05]. Post-hoc pairwise mean comparisons revealed that happy faces elicited greater forward body movements than neutral faces [Z = 2.56, p<.05], whereas there were no differences between happy and pain faces [Z = 1.84, NS], or between pain and neutral faces [Z = .89, NS] (Figure 3).

No significant effects of facial expressions were found on the amplitudes of either backward sway movements [χ^2^(2) = 2.15, NS] or body sways in the medial-lateral axis [χ^2^(2) = 2.15, NS].

Kendall's tau-b (τ) coefficients were computed between amplitude of body sway movements and pleasantness (Figure S1), arousal (Figure S2) and scores from the Interpersonal Reactivity Index (IRI) (Figures S3, S4, S5, S6) separated for each facial expression. Regarding happy faces, increased pleasantness was significantly correlated with reduced amplitude of backward movements [τ(40) = −.30, p<.05], whereas increased forward movements of the body were correlated with high scores on the Empathic Concern (EC) [τ(40) = .25, p<.05] and Personal Distress (PD) [τ(40) = .23, p<.05] subscales. Regarding pain faces, increased amplitude of body sway was correlated with high unpleasantness [τ(40) = −.28, p<.05], as well as high scores on the Empathic Concern (EC) [τ(40) = .24, p<.05] and Personal Distress (PD) [τ(40) = .25, p<.05] subscales. In addition, high scores on Personal Distress were also correlated with increased forward movements elicited by pain faces [τ(40) = .23, p<.05]. No significant correlations were found for neutral faces.

## Discussion

The aim of the present study was to evaluate approach-avoidance behaviors elicited by the observation of facial expressions of pain. For this purpose, we compared changes on postural sway of the body when participants were standing and observing video clips of pain, happy and neutral faces. As it was expected, participants rated pain faces as the most unpleasant stimuli followed by neutral and happy faces. On the other hand, we observed that both happy and pain faces elicited greater postural sway of the body in the anterior–posterior axis compared with neutral faces. No significant effects due to facial expressions were observed in postural sway of the body in the medial-lateral axis. Finally, unpleasantness was significantly correlated with increased amplitude of body sway movements of the body elicited by pain faces. In a similar way, high scores on the Empathic Concern subscale of the Interpersonal Reactivity Index (IRI) were correlated with increased body sway movements in the anterior-posterior axis and, in particular, with increased amplitudes of forward body movements.

The fact that happy faces elicited increased amplitudes of forward body movements during standing is in agreement with previous studies showing that happy faces can induce approaching and cooperation responses [14], [15], [17]. Thus, for instance, previous studies have shown that happy faces elicited the fastest responses when participants were asked to push or to pull a joystick in response to pictures of different facial emotional expressions [15]. A similar result was also obtained when participants were asked to make whole-body forward (approach) or backward (avoidance) steps in response to happy and angry faces [27]. These data indicated that participants needed less time to initiate a forward step towards a smiling face than towards an angry face, suggesting that approaching a friendly face may invite to physical contact such as a hug or a handshake, whereas approaching an angry face may expose oneself to potential physical and verbal abuse [27]. By contrast, a previous study of the same group revealed that there were no differences between happy and neutral faces on body sway when static pictures of facial expressions were used as stimuli [28]. In our study, we found that participants displayed greater forward body sway when they were viewing dynamic facial expressions of happiness in comparison with neutral faces. Thus, our finding seem to suggest that this approaching tendency characterized by small and spontaneous forward sway movements of the body could be elicited in response to dynamically and, hence, more realistic movement of the happy face.

In addition, we observed that although pain faces were rated as more unpleasant than happy faces, they elicited similar amplitudes of forward sway movements than happy faces, together with increased amplitudes of body sway movements as compared to neutral faces. These findings are in disagreement with previous research on postural sway and body movements elicited by unpleasant stimuli (such as angry faces, threat or mutilation images). Thus, for instance, it has been reported that fear or threat pictures may induce reduced sway (freezing responses) [28], [35], as well as increased backward movements (avoidance responses) [27]. According with animal research and Lang's bio-informational model [1], these behavioral reactions mimics the post-encounter stage of threat that is typically observed in animals, and that involves a sequence of freeze-flight-fight responses. By contrast, our findings seem to indicate that the effects of pain faces on postural standing are different from those observed with other unpleasant stimuli such as threat, anger and fear stimuli. Indeed, pain faces are usually perceived as unpleasant and activating [29], [32], and even more unpleasant and arousing than anger faces for all intensity levels of the facial expression [32]. Furthermore, it has been shown that amplitudes of the visual evoked potentials elicited by facial expressions of pain and anger are significantly different [32], suggesting the involvement of different brain mechanisms during the processing and recognition of facial expressions of pain and anger in healthy volunteers. Thus, taking into account that accurate perception of other's pain may be considered as a relevant cue for delivering effective care and social support when in pain, our finding that viewing pain faces elicited greater amplitudes of forward sway movements than neutral faces (as it happens with happy faces in the current study) could be interpreted as an approaching behavioral tendency. Additionally, the significant correlations of unpleasantness ratings or empathic measures (such as Personal Distress and Empathic Concern) with increased body sway and forward movements elicited by pain faces seem to further support this interpretation.

There are, however, some limitations of our study that merit further consideration. First, pain and happy faces differed in pleasantness ratings, but they were similar in arousal ratings, and therefore the specific influence of this affective dimension on postural standing should be further clarified. A second shortcoming of the study was that our sample consisted only of women. We decided to use only female participants because significant differences between men and women on postural responses to emotional faces have been previously reported [13], [36]. Nevertheless, future research should investigate if gender may also play a significant role on changes of postural standing when observing facial expression of pain in others. Third, although some correlations between body sway and psychological variables were significant, they were small and no correction for multiple comparisons were performed. Therefore, these findings should be taken with caution and considered as exploratory. And finally, our video recording and analysis methods represent novel approaches that have never been used before for quantitative measurement of changes in postural sway. Further investigation should compare this method with standardized posturographic methods in order to check the reliability of this technique.

## Supporting Information

Figure S1 Scatterplots of pleasantness ratings versus body sway parameters (amplitude of body sway in the anterior–posterior axis, amplitude of forward movements, amplitude of backward movements) for all three facial expressions (happy, pain and neutral).
(TIF)

Figure S2 Scatterplots of arousal ratings versus body sway parameters (amplitude of body sway in the anterior–posterior axis, amplitude of forward movements, amplitude of backward movements) for all three facial expressions (happy, pain and neutral).
(TIF)

Figure S3 Scatterplots of Perspective Taking (PT) scores versus body sway parameters (amplitude of body sway in the anterior–posterior axis, amplitude of forward movements, amplitude of backward movements) for all three facial expressions (happy, pain and neutral).
(TIF)

Figure S4 Scatterplots of Fantasy (FS) scores versus body sway parameters (amplitude of body sway in the anterior–posterior axis, amplitude of forward movements, amplitude of backward movements) for all three facial expressions (happy, pain and neutral).
(TIF)

Figure S5 Scatterplots of Empathic Concern (EC) scores versus body sway parameters (amplitude of body sway in the anterior–posterior axis, amplitude of forward movements, amplitude of backward movements) for all three facial expressions (happy, pain and neutral).
(TIF)

Figure S6 Scatterplots of Personal Distress (PD) scores versus body sway parameters (amplitude of body sway in the anterior–posterior axis, amplitude of forward movements, amplitude of backward movements) for all three facial expressions (happy, pain and neutral).
(TIF)
